# Co-Cultivation of Fungal and Microalgal Cells as an Efficient System for Harvesting Microalgal Cells, Lipid Production and Wastewater Treatment

**DOI:** 10.1371/journal.pone.0113497

**Published:** 2014-11-24

**Authors:** Digby Wrede, Mohamed Taha, Ana F. Miranda, Krishna Kadali, Trevor Stevenson, Andrew S. Ball, Aidyn Mouradov

**Affiliations:** Royal Melbourne Institute of Technology University, School of Applied Sciences, 3083 Bundoora, VIC, Australia; University of Nebraska-Lincoln, United States of America

## Abstract

The challenges which the large scale microalgal industry is facing are associated with the high cost of key operations such as harvesting, nutrient supply and oil extraction. The high-energy input for harvesting makes current commercial microalgal biodiesel production economically unfeasible and can account for up to 50% of the total cost of biofuel production. Co-cultivation of fungal and microalgal cells is getting increasing attention because of high efficiency of bio-flocculation of microalgal cells with no requirement for added chemicals and low energy inputs. Moreover, some fungal and microalgal strains are well known for their exceptional ability to purify wastewater, generating biomass that represents a renewable and sustainable feedstock for biofuel production. We have screened the flocculation efficiency of the filamentous fungus *A. fumigatus* against 11 microalgae representing freshwater, marine, small (5 µm), large (over 300 µm), heterotrophic, photoautotrophic, motile and non-motile strains. Some of the strains are commercially used for biofuel production. Lipid production and composition were analysed in fungal-algal pellets grown on media containing alternative carbon, nitrogen and phosphorus sources contained in wheat straw and swine wastewater, respectively. Co-cultivation of algae and *A. fumigatus* cells showed additive and synergistic effects on biomass production, lipid yield and wastewater bioremediation efficiency. Analysis of fungal-algal pellet's fatty acids composition suggested that it can be tailored and optimised through co-cultivating different algae and fungi without the need for genetic modification.

## Introduction

Although substantial efforts are being made worldwide to produce renewable biofuels, significant challenges still need to be overcome before microalgal–based biofuel production becomes cost-effective and can impact the world's supply of transport fuel [1], [2], [3], [4], [5], [6]. Optimising algal harvesting/dewatering technologies is a significant challenge that needs to be addressed for the development of a cost-effective large scale algal biofuel. Additional challenges include a sustainable nutrient supply and efficient, cost effective technologies for lipid extraction.

Harvesting can account for up to 50% of the total cost of biodiesel production and is not economically viable for the microalgal industry because of increased energy requirements and the addition of chemicals (for reviews see [7], [8], [9], [10], [11], [12], [13], [14]). The main techniques used for harvesting algal cells include centrifugation, filtration, flocculation, gravity sedimentation and flotation [15], [16], [14], [17], [18]. Filtration has been shown to be highly efficient, but only for the large multicellular microalgae such as *Coelastrum proboscideum* and *Spirulina platensis* and frequent filter replacement makes this method uneconomical [14], [19]. Moreover, this process is slow, although, processing speed can be increased through the addition of flocculants [20]. Centrifugation is an efficient technology and can harvest about 90% of the microalgae; however this comes with a high energy input cost, especially with a low value product such as biofuel [21]. The floatation method includes air or gas bubbles or flocculants attach to the algal cells carrying them to the surface [22], [23] [14]. Recently Garg et al. (2014) showed that the recovery of marine microalga *Tetraselmis* sp. can be increased up to 97.4% using improved froth floatation performance [24]. Flocculation is the process by which the algae forms clumps, pellets or pellet like structures called flocs. The negatively charged microalgal surfaces prevent their self-flocculation under normal growth conditions [25], [26], [14], [27]. In general, flocculation technology addresses this issue by neutralizing or reducing microalgal surface charge using chemical flocculants (inorganic and organic), biological organisms or using an electrical impulse [28]. These methodologies, however, are not universally successful and do not work for all microalgae strains [28], [29]. Flocculation can be induced by biological organisms such as bacteria and fungi [30], [21], [31], [32]. An efficient bacterial bioflocculant has been isolated from the autoflocculating *Scenedesmus* spp and *Chlorella vulgaris* (*C. vulgaris*) microalgae when they were grown in wastewater [33], [34], [35], [31]. Gram-positive bacteria *Solibacillus silvestris and Bacillus* sp also showed a flocculation efficiency of up to 90% with the marine microalgae *Nannochloropsis oceanica* [36], [32].

Filamentous fungi represent attractive bioflocculating agents because of their self-pelletization and high microalgal trapping efficiencies. Fungal self-pelletization has been observed for numerous filamentous strains and can be explained by coagulative and non-coagulative mechanisms [37], [38], [39], [15], [40]. The coagulative mechanism observed in representatives of *Aspergillus spp*, *Basidiomycete spp*, *Phanerochaete spp* involves spore coagulation leading to developments of aggregates/pellets. As a result fungi produce dense spherical aggregates [15], [37]. The non-coagulative mechanism involves spores germinating into hyphae, which then intertwine into pellets. Representatives of *Rhizopus spp, Mucor spp* and *Penicillium spp* display the non-coagulative mechanism [15] [37]. Fungal assisted harvesting technology does not require addition of chemicals or inputs of energy and has been shown to be an efficient for one microalgal strain, *C. vulgaris* [15], [37], [41], [42]. If this technology can be applied to the commercially important freshwater and seawater algal species used for biodiesel production, the procedure can offer a solution to at least one of the major problems associated with the energy-intensive and costly harvesting processes.

Natural symbiosis between fungi and algae or fungi and cyanobacteria, also known as lichens, have existed since plants evolved from green algae more than 400 million years ago and covering 6% of Earth's land surface [43] (Figure S1). In this mutually beneficial symbiosis, fungi consume the photosynthetic carbon provided by the algae as sugars and nutrients; in return the fungus provides protection to the algae by retaining water and serving as a larger capture area for mineral nutrients [44]. This suggests that lichen can at least partially represent a self-sufficient symbiotic association. Discovery of lignin and cellulose degrading enzymes secreted by lichens suggests their saprophytic activities, at least in the immediate vicinity of the thallus. These activities can be beneficial in periods of limited microalgal photosynthetic activity at the time when lichens are covered by snow or leaves [45].

In our work for the first time filamentous fungi *A. fumigatus* was tested for its flocculation efficiency against a 11 microalgal strains representing, photoautotrophic and heterotrophic, freshwater and marine, unicellular and multicellular, small (3 µm) and giant (300 µm), motile and non-motile strains. Some of these strains are commercially used for biofuel production. The lipid levels and composition were analysed for fungal-algal pellets grown on glucose and alternative carbon sources, in freshwater, seawater and wastewater containing media. Our research showed that pelletization has additive and synergistic effects on the level and composition of lipids and on the efficiency of wastewater treatment.

## Materials and Methods

### *A. fumigatus* isolation

*A. fumigatus* isolates were sourced from an areas around piles of straw located at either at Flinders University (Adelaide, Australia, GPS position: 35°01′28.02″S, 138°34′16.82″E) or at RMIT University, Bundoora campus, (Melbourne,Australia, GPS position: 37°40′37.20″S 145°04′19.52″E). Both of these sites both allowed sampling without specific permission. The field studies did not involve endangered or protected species.

These samples were kept in zip lock plastic bags and stored at −20°C for further investigation. The collected samples were serially diluted (10∶1 to 10∶6) using phosphate buffer saline (0.1 M) and an aliquot (150 µl) of each dilution was spread onto BH agar plates [46]. These plates were incubated for 6 days at 30°C and 55°C for mesophilic and thermophilic fungi respectively. For fungal isolations, an antibiotic solution of 0.015 g/l of tetracycline (dissolved in sterilized Milli-Q water, filtered through a sterile 0.22 µm filter) was added to the media. Following isolation fungi were re-streaked until purified. All cultures were stored in 25% of glycerol at −80°C.

### Genotyping

The identification of the fungal strain was based on nucleotide sequence analysis of the internal transcribed space (ITS) region. Genomic DNA was extracted as described by [47], [48]. The ITS1 region was amplified by PCR with primers ITS1: TCCGTAGGTGAACCTGCGG and ITS2: GCTGCG TTCTTCATCGATGC [47]. Satisfactory 16S rDNA sequences were codon aligned, and compared with published reference strains in the National Centre of Biotechnology Information. An alignment between the query and reference sequences of more than 95% denoted a positive match. Confirmatory phylogenetic reconstruction was also performed using standard bioinformatic software such as the PAUP (Sinauer Associates Inc., Sunderland, Massachusetts),

### Preparation for seed fungal spores

For activation the stored spores were grown at 25°C for five days on plates with Potato Dextrose Broth (PDB) containing 20 g/l glucose. Sterile water (10 ml) was added to harvest the spores and the spore solution was used as the inoculation for the co-culture after the number of spores in the solution were counted.

### Microalgal strains

Microalgal strains, their sources, characteristics and growth media are described in Table S1. All strains were grown axenically in growth media suggested by manufactures (Table S1). *Thraustochytrid* sp was grown under heterotrophic conditions (10 g/l glucose), as suggested by the American Type Culture Collection (ATCC). Autotrophic strains were grown under constant light (200 µmol m^−2^ s^−1^), shaking at 150 rpm at 25°C. Growth rates were analysed by (i) counting the cell numbers using a TC10 Automated Cell Counter (BioRad), (ii) measuring OD_660_ for *Thraustochytrid* sp and OD_750_ for other strains, (iii) by determining the concentration of chlorophyll A+B using a POLARstar Omega Multi-Mode Microplate Reader with Fluorescent Polarization (BMG LABTECH) and (iv) by biomass production. Chlorophyll was extracted with ethanol and extinctions at 649, 665, and 750 nm were determined. Chlorophyll concentration (µg/ml) was calculated using the equation Chl (µg/mL) = [6.1×(E665–E750)+20.04 (E649–E750)], K, where E is extinction at the corresponding wavelength; K is the dilution factor and 6.1 and 20.04 are extinction coefficients [49]. For biomass analysis microalgal cultures were centrifuged at 6000 *g* and then was washed twice with sterilized water and centrifuged again and dried at 65°C.

### Pelletization and fungal-assisted flocculation

To achieve pelletization spore solutions (1.5–2.0×10^7^ spores/l) were cultivated at 28°C in liquid fungal growth broth (FGB): 3 g/l peptone, 0.6 g/L KH_2_PO_4_, 0.001 g/l ZnSO_4_, 0.4 g/l K_2_HPO_4_, 0.005 g/l FeSO_4_, 0.5 g/l MnSO_4_, 0.5 g/l MgSO_4_. As a carbon source we used 20 g/l glucose (*A. fumigatus*/GLU) or 1% acid pre-treated wheat straw (TWS, *A. fumigatus*/TWS) with a shaking speed of 150 rpm for 72 h. Pellets were precipitated and growth medium was removed. Algal cultures were precipitated, washed and resuspended to achieve a final concentration of 5–8×10^8^ cell/mL and added to fungal pellets. The fungal-algal mixtures were shaken at 150 rpm for 48 h under constant light (200 µmol m^−2^ s^−1^) at 25°C. Fungal and algal mono-cultures were also grown for 48 h as controls. All of the experiments were biologically replicated at least three times. Cell number, biomass, OD_660/750_ and chlorophyll concentrations were measured at time 0, 24 h and 48 h. For flocculation efficiency (FE) analysis algal samples were analysed 3 mins after stopping rotation (Figure S2). FE was calculated based on changes in OD, cell numbers, biomass and in chlorophyll concentrations of uncaptured algal cells in the co-cultivation media at time 0, 24 and 48 h later according to the formula: FE% = [(A–B)/A]×100 where A = OD, cell number, biomass, chlorophyll concentration at time 0; B = OD, cell number, biomass, chlorophyll concentration after 24 h or 48 h. The morphology of the fungal and algal cells and co-cultivation pellets was observed under bright field conditions using a Leica DM 2500 with an attached camera (Leica DFC 310 FX).

A half maximal flocculation efficiency (FE_50_) was measured to find a minimum amount of *A. fumigatus* required to harvest 50% of microalgal cells. Three concentrations of *A. fumigatus*/GLU and *A. fumigatus*/TWS fungal pellets (0.2–0.5 cm in diameter) were washed and mixed with 100 ml of microalgal suspension (5–8×10^8^ cell/ml): 1) 10%, as 10 ml pellets suspension (150±12 g wet weight, 0.12±0.03 g dry weight); 2) 5%, as 5 ml pellets suspension (75±9 g wet weight, 0.07±0.02 g dry weight); 3) 2.5%, as 2.5 ml pellets suspension (35±6 g wet pellets, 0.04±0.01 g dry weight). FE_50_ (g DW/l) was calculated by correlating the dry weight of the pellets with the obtained FE for each algal strain.

### Acid pre-treatment of wheat straw

One gram of fine powder (approximately 1 mm sin size) of dry wheat straw was mixed with 1 M sulphuric acid and autoclaved for 10 min at 121°C, allowed to cool, filtered through Whatman No. 1 filter paper, then washed with 0.1 M sodium hydroxide followed by washing 10 times with sterile water. The powder was dried at 80°C and added to the media to a final concentration of 1%.

### Nile Red staining

For Nile Red staining the algal cells, fungal cells and co-cultivated pellets were collected by centrifugation and re-suspended in 1 ml of 20% DMSO containing 5 µl of Nile Red stock solution (0.10 mg/ml of Nile Red dissolved in acetone) and incubated at 50°C with shaking at 150 rpm for 5 min. The stained pellets were then subjected to fluorescent microscopy analysis to observe the formation of lipid droplets in the co-cultivated cells using a Leica DM 2500 with an attached camera (Leica DFC 310 FX) and Nile-Red filter, excitation at 543 nm, emission 555–650 nm.

### Wastewater treatment

The anaerobically digested swine lagoon wastewater (ASW) was provided by Dr J Hill, Termes Consulting Ltd, Melbourne. Swine wastewater was treated anaerobically. Wastewater samples were centrifuged to remove large particles, filtered through Whatman filter paper, autoclaved at 121°C, allowed to cool to room temperature, and stored at 4°C. The concentrations of NH_4_^+^–N and PO_4_^−3^-P in the ASW were 680.7 mg/l and 145.7 mg/l, respectively. The concentration of other inorganic nitrogen in the wastewater, such as NO_3_^−^-N was very low and not reported. Wastewater was diluted to 25% and 10% with sterile seawater for experiments with *Thraustochytrid* sp and *T. chuii*. The *A. fumigatus*/PDB - algal pellets were harvested by filtration and 200 wet pellets were added to 250 ml of wastewater (approximately, 1 g/l DW). The mixtures were shaken at 150 rpm for 48 h. Samples of growth media were analyzed for ammonium, nitrate and phosphate concentrations using an ion chromatography system Dionex ICS-1100 (Thermo Scientific, USA).

### Lipid yield and fatty acid profile analysis

Extraction and analysis of lipid yield and FAME composition analysis of algal, fungal and fungal-algal pellets were performed using a method previously described [50].

### Statistical analysis

All experiments in this study were conducted in triplicate. The experimental data were subjected to one-way analysis of variance (ANOVA) as implemented in the GraphPad InStat 3 statistics platform. Tukey simultaneous tests were conducted to determine the statistical differences between treatments. In order to ascertain that the observed variations in growth rates, efficiency of nutrients uptake and the yield of pyrolysis products were statistically significant, the probability (p) values were determined. A 95% confidence level (p≤0.05) was applied for all analyses.

## Results

### Flocculation of microalgal cells by *A. fumigatus*

**Figure 1:**
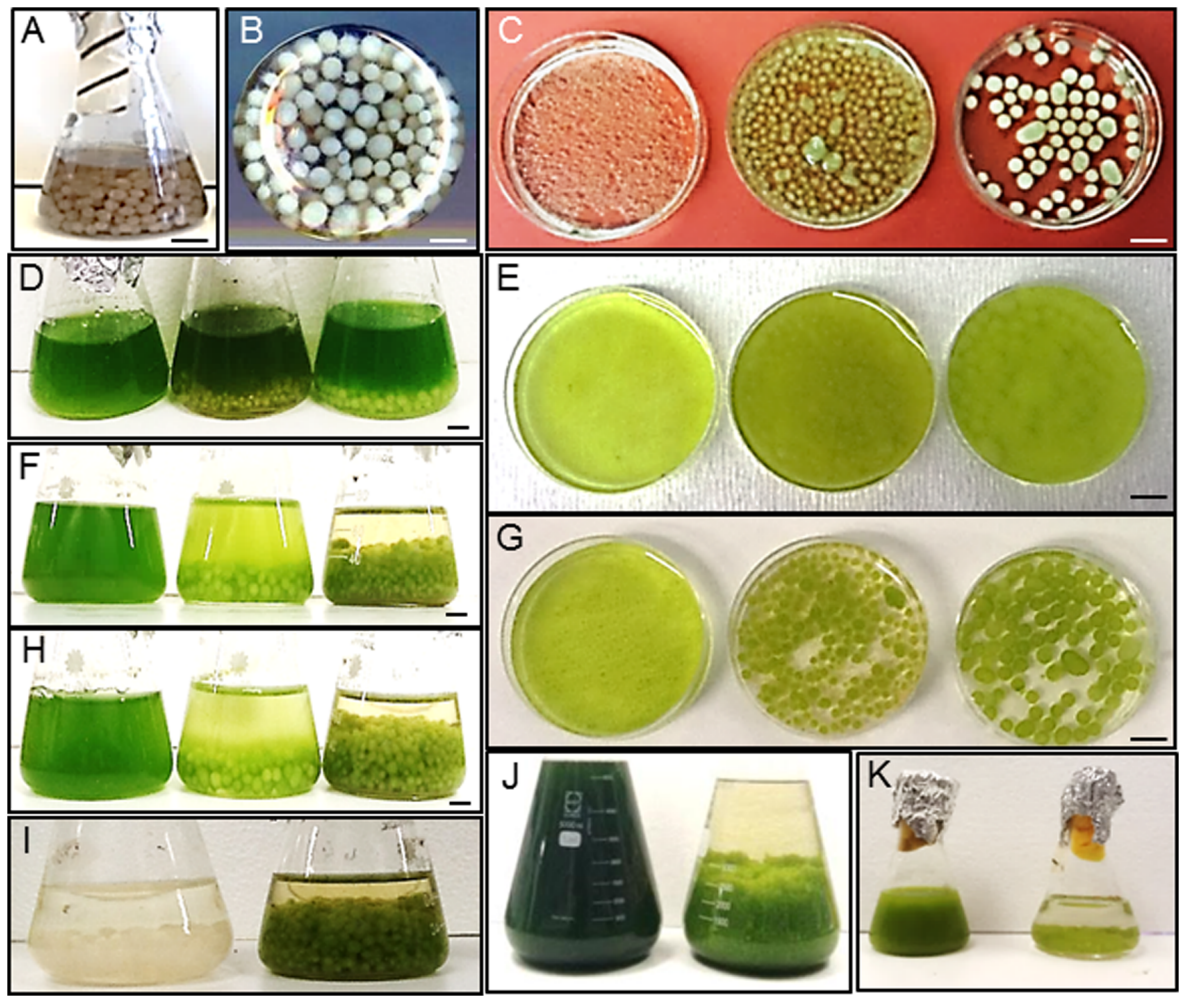
Flocculation of microalgal strains by *A. fumigatus*. A, B) *A. fumigatus* pellets; C) *A. fumigatus* pellets grown on carbon-free FGB (*A. fumigatus*/FGB, left), FGB with 1% TWS (*A. fumigatus*/TWS, middle) and FGB with 20% glucose (*A. fumigatus*/GLU, right); D–H) Flocculation of *P. subcapitata by A. fumigatus*/FGB (left), *A. fumigatus*/TWS, (middle) and *A. fumigatus*/GLU (right). Time, 0 (D, E); 24 h (F) and 48 h (G, H); I) *A. fumigatus* pellets grown in mono-culture in algal media for 48 h (control, left) and *A. fumigatus* pellets grown with *P. subcapitata* in algal media for 48 h (right); J) Flocculation of *T. chuii:* Time 0 (left), 48 h (right); K) Flocculation of *N. oculata:* time 0 (left), 48 h (right).

The cultures of filamentous fungi *A. fumigatus* produced dense spherical pellets, approximately 2–5 mm in size, when grown on FGB containing glucose (20 g/l) under 150 rpm rotation (*A. fumigatus*/GLU) (Figure 1A). To assess flocculation efficiency *A. fumigatus*/GLU pellets were mixed with high cell density cultures of microalgal cultures (5–8×10^8^ cell/ml) representing fresh water and marine strains (Figure 1). The list of freshwater algal strains includes: *Chlorella vulgaris (C. vulgaris), Chlamydomonas reinhardtii (C. reinhardtii), Pseudokirchneriella subcapitata (P. subcapitata)* and *Scenedesmus quadricauda (S. quadricauda)*. The marine microalgae tested include *Thraustochytrid* sp, *Dunaliella tertiolecta (D. tertriolecta), Dunaliella salina (D. salina), Nannochloropsis oculata (N. oculata), Nannochloris oculata (Nl. oculata)*, *Tetraselmis chuii (T. chuii)* and the dinoflagellate, *Pyrocystis lunula* (*P. lunula*). Phenotypic characteristics of microalgal strains are shown in Table S1.

**Figure 2:**
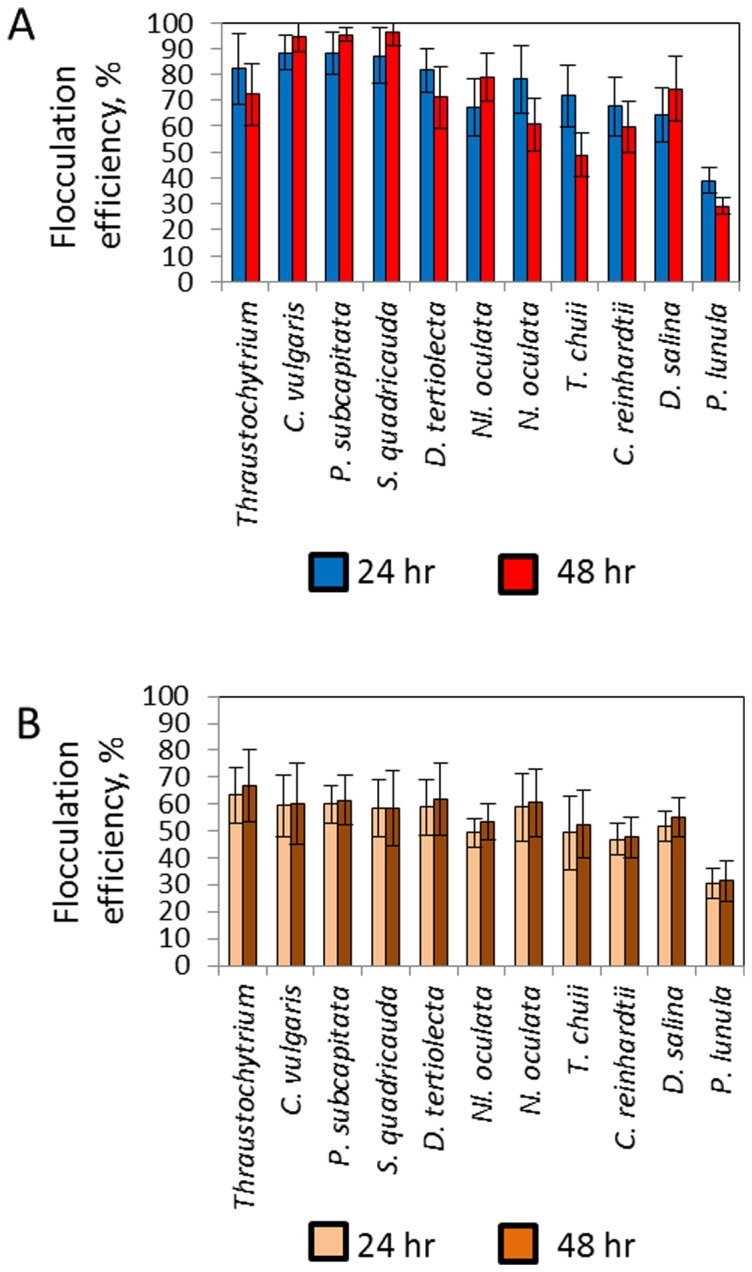
Flocculation efficiency of microalgal cells by *A. fumigatus*. Flocculation of microalgal cells by *A. fumigatus*/GLU pellets; (B) *A. fumigatus*/TWS pellets. Flocculation efficiency of *A. fumigatus* with all microalgal strains showed significance levels, p<0.01.

**Figure 3:**
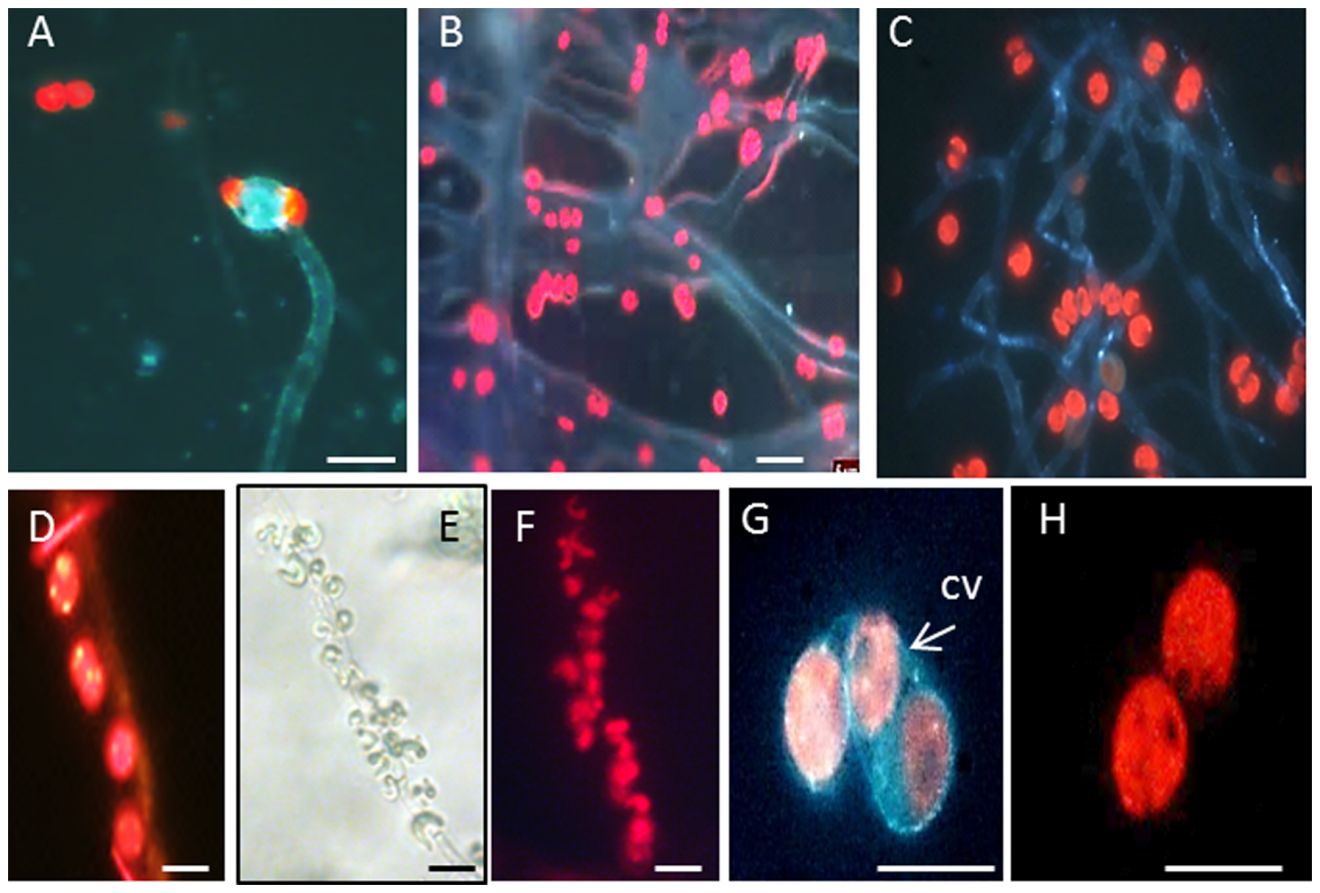
Microscopic analysis of *A. fumigatus*-miroalgal interactions. Images of miroalgal cells attached to *A. fumigatus* filaments. A, B) *D. tetrioletta*; C,D) *T. chuii*; E,F): *P. subcapitata*; G,H) *T. chuii*. A,B,C,D,F,G,H: UV light images. Red spots represent chloroplast's fluorescence; E) bright-field image CV: cell walls. Scale = 20 µm.

*A. fumigatus* showed up to 90% flocculation after first 24 h of co-cultivation with no obvious differences in flocculation efficiency between freshwater and seawater, motile and non-motile species (Figure 2A). The largest algae (dinoflagellate) assessed, *P. lunula* (over 300 µm), showed the lowest rate of flocculation. For some of the algal representatives the concentration of uncaptured microalgal cells in media were increased after 24 h (shown as a decrease in flocculation efficiency), which can be explained by their release from the fungal filaments and/or their independent growth in the media. Half maximal flocculation efficiency (FE_50_) as a minimum amount of *A. fumigatus* cells required to harvest 50% of microalgal cells is shown on Table 1. Detailed microscopic analysis of the *A. fumigatus*-algal pellets showed that microalgal cells can tightly bind to the fungal filaments (Figure S3 and Figure 3). For some microalgal strains such as *T. chuii* a clear lack of cell walls was observed for cells entrapped in fungal filaments.

**Table 1:** Half maximal flocculation efficiency (FE_50_) of *A. fumigatus* grown on glucose and TSW.

Species	*A. fumigatus*/GLU		*A. fumigatus*/TSW	
	FE_50_ (gDW/l)	R^2^	FE_50_ (gDW/l)	R^2^
*Thraustochytrium* sp	6.2±1.2	0.89	7.1±2.2	0.85
*C. vulgaris*	4.9±1.1	0.82	7.28±2.3	0.86
*P. subcapitata*	4.5±1.1	0.86	7.2±2.2	0.86
*S. quadricauda*	4.6±1.2	0.82	7.2±1.8	0.81
*D. tertiolecta*	6.1±1.4	0.85	6.7±1.2	0.82
*Nl. oculata*	5.5±1.4	0.85	6.3±1.6	0.85
*N. oculata*	7.5±2.1	0.84	6.9±2.2	0.87
*T. chuii*	9.3±3.0	0.78	6.3±1.7	0.81
*C. reinhardtii*	7.4±2.8	0.81	6.9±2.2	0.78
*D. salina*	6.9±2.4	0.78	7.2±2.3	0.78
*P. lunula*	20.1±6.4	0.81	24.6±7.1	0.84

To test the efficiency of algal flocculation by *A. fumigatus* grown on alternative carbon source fungal spores were grown on carbon-free broth containing 1% TWS (*A. fumigatus*/TWS). Compared to *A. fumigatus*/GLU pellets *A. fumigatus*/TWS produced smaller size pellets (approximately 2–4 mm size) which, however, were significantly larger than pellets produced in media containing no added carbon sources (approximately 1 mm in size) (Figure 1C). As a preliminary step we wanted to assess the potential anti-algal effect of TWS digested by *A. fumigatus*. For this we grew algal cells in the presence of 5% and 20% media collected 72 h after incubation of *A. fumigatus* with TWS. Suppression of algal growth was observed in the presence of 20% added media (Figure S4). To avoid this effect *A. fumigatus*/TWS pellets were washed before mixing with microalgal cultures. Figures 1 and 2B shows flocculation rates of the *A. fumigatus*/TWS pellets which were found to be lower than the flocculation rate of *A. fumigatus*/GLU pellets (Figure 2A). This can be explained by the effect of residual amount of chemicals in the growth media as well as by digestion of algal cell walls by the cocktail of hydrolytic enzymes secreted from *A. fumigatus* in the presence of TWS. No significant increase in the numbers of uncaptured algal cells was detected in the media after 24 h of co-cultivation. As expected FE_50_ for *A. fumigatus*/TWS pellets showed higher values compare to FE_50_ observed for the flocculation of microalgal strains by *A. fumigatus*/GLU pellets (Table 1).

### Lipid production in *A. fumigatus*-microalgal pellets

Mono-cultured *A. fumigatus*/GLU pellets before mixing with microalgal cultures showed a lipid content of 12% of its dry weight (DW) biomass and a lipid yield of 8.1 mg/l (Time 0, Table 2). Not surprisingly, *A. fumigatus*/TWS pellets showed significantly lower lipid content, 2.9% of DW with a lipid yield of 1.7 mg/l (Time 0). Mono-cultured microalgal strains showed a wide range of lipid concentrations with the heterotrophic *Thraustochytrid* sp showing highest levels, up to 38% of its DW biomass.

**Table 2:** Biomass and lipids production in *A. fumigatus* and microalgal strains grown in mono-cultures and co-cultures.

	Fungi/Microalgae monocultures, 0 h			Fungi/Microalgae monocultures, 48 h			Microalgae+*A. fumigatus/TWS*, co-cultures, 48 h			Microalgae+*A. fumigatus/GLU*, co-cultures, 48 h		
Species	Biomass (g/l)	Lipids (%)	Lipid yield (mg/l)	Biomass (g/l)	Lipids (%)	Lipid yield (mg/l)	Biomass (g/l)	Lipids (%)	Lipid yield (mg/l)	Biomass (g/l)	Lipids (%)	Lipid yield (mg/l)
***A. fumigatus***												
***A. fumigatus/TWS***	0.07±0.01	2.91±0.2	1.71±0.7	0.13±0.01	3.69±0.1	4.11±0.9	NA	NA	NA	NA	NA	NA
***A. fumigatus/GLU***	0.08±0.01	12.02±3.6	8.12±1.9	0.20±0.03	12.10±3.1	25.46±4.1	NA	NA	NA	NA	NA	NA
**Freshwater microalgae**												
***C. vulgaris***	0.07±0.01	22.20±2.7	14.59±1.7	0.20±0.02	23.20±2.8	45.74±5.5	0.47±0.0*	9.85±2.1	46.2±5.5	0.80±0.1**	18.35±2.2	146.07±17.6**
***P. subcapitata***	0.06±0.01	16.14±3.1	10.74±1.9	0.29±0.04	24.91±3.0	72.42±8.7	0.56±0.0*	5.21±1.7	30.76±4.1	0.75±0.1**	10.21±2.4	78.45±18.2
***C. reinhardtii***	0.06±0.01	7.37±0.8	4.68±0.5	0.27±0.03	5.67±0.6	15.35±1.8	0.54±0.1*	3.81±0.5	25.99±3.1#	0.85±0.1**	8.59±1.0	73.35±8.8*
***S. quadricauda***	0.05±0.01	6.87±0.8	3.68±0.4	0.32±0.04	6.67±0.8	21.39±2.8	0.59±0.07#	3.96±1.2	30.86±5.4#	0.62±0.1#	7.86±0.9	49.01±5.9#
**Marine microalgae**												
***Thraustochytrium sp***	0.08±0.03	38.20±9.4	32.23±7.6	0.28±0.08	36.20±8.8	101.70±25.6	0.71±0.2*	16.35±4.8	125.34±36.3**	1.06±0.2**	23.35±4.1	245.96±36.1**
***T. chuii***	0.07±0.03	12.34±5.6	9.26±3.0	0.20±0.04	11.80±3.5	23.15±5.2	0.31±0.7	6.3±3.7	20.69±8.2	0.59±0.1*	11.21±4.1	67.67±14.1#
***D. tertrioleta***	0.05±0.01	16.12±1.9	8.84±1.0	0.16±0.01	12.22±1.4	20.11±2.4	0.43±0.1*	6.86±0.9	30.80±4.0#	0.50±0.1*	13.86±1.6	69.57±8.3*
***Nl. oculata***	0.03±0.01	11.88±1.4	4.18±0.5	0.11±0.01	11.71±1.4	12.34±1.4	0.38±0.1*	4.60±0.8	17.80±3.0	0.49±0.1*	9.60±1.1	47.29±5.7#
***N. oculata***	0.06±0.01	27.40±3.3	18.48±2.2	0.20±0.02	25.10±3.0	50.79±6.1	0.47±0.1*	10.30±1.2	48.41±5.8	0.60±0.1*	18.30±2.2	109.31±13.1**
***P. lunula***	0.07±0.02	7.30±2.4	4.92±1.2	0.27±0.03	7.7±2.1	20.97±6.1	0.40±0.1	4.30±1.2	18.41±4.1	0.51±0.1#	8.10±2.6	41.68±8.8
***D. salina***	0.04±0.01	12.83±1.5	5.19±0.6	0.12±0.01	12.58±1.5	15.26±1.8	0.39±0.1#	6.42±0.7	25.02±3.0#	0.58±0.1*	10.94±1.3	63.10±7.6*

*S*ynergistic effects of *A. fumigatus*/microalgal co-cultures with significance levels: *p<0.05; **p<0.01.

*A*dditive effect of *A. fumigatus*/microalgal co-cultures: #.

Lipid production in the fungal-algal pellets showed complex profiles reflecting at least three main factors: (i) total biomass production, (ii) lipid concentrations in fungal and algal cells before and during co-cultivation and (iii) the harvesting efficiencies of algal cells by *A. fumigatus* pellets. After 48 h of co-culture of *A. fumigatus*/GLU with oleaginous microalgae (lipid concentration >10% of DW) lipid concentration in pellets were found lower than in mono-cultured algae, but similar or higher to that seen in mono-cultured *A. fumigatus*. The total lipid yields (mg/l) in most of pellets were also found similar or higher than the additive lipid content of mono-cultured algal and fungal strains (Table 2). Co-cultivation of *A. fumigatus/GLU* pellets with *C. vulgaris*, *C. reinhardtii*, *Thraustochytrid* sp, *D. tertriolecta*, *D. salina* and *N. oculata* showed synergistic effect on total lipid yields.

Lipid concentrations of *A. fumigatus*/TWS pellets with all microalgal strains were found to be lower than in mono-cultured algae, but similar or higher than in mono-cultured *A. fumigatus*/TWS (Table 2). The lipid yields (mg/l) after co-culturing *A. fumigatus*/TWS with most of the microalgal strains, was similar or lower than the additive amount of total lipid yields.

### Fatty acid composition in fungal-microalgal pellets

**Figure 4:**
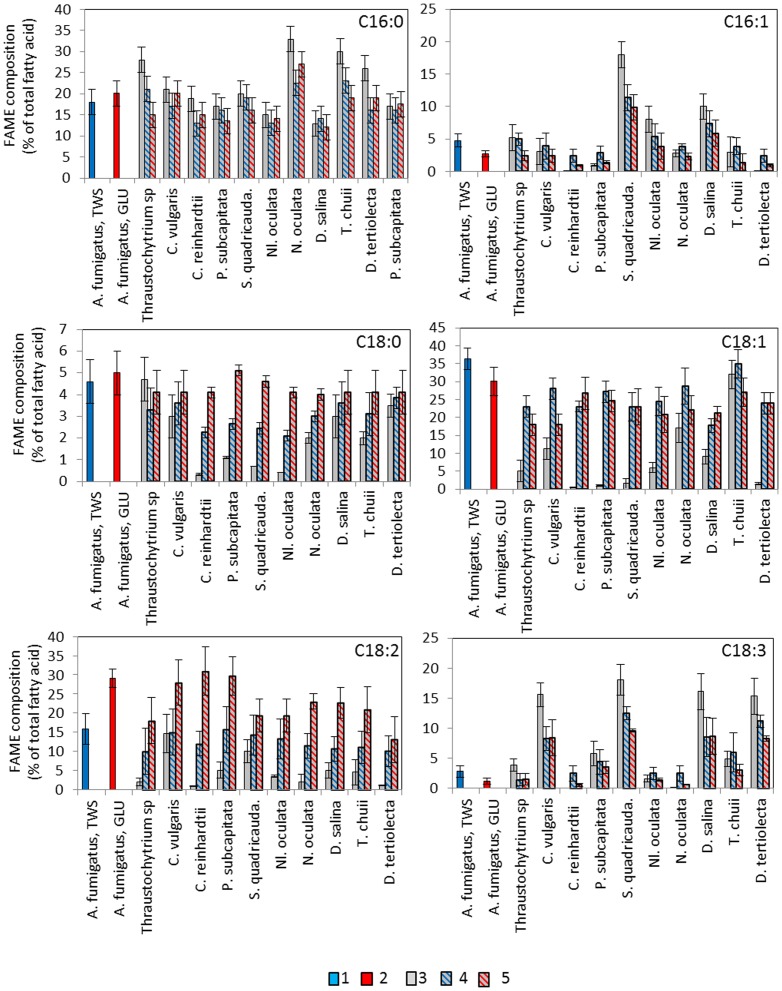
Fatty acids composition of *A. fumigatus*, microalgae and *A. fumigatus*-microalgal pellets. 1) *A. fumigatus*/TWS; 2) *A. fumigatus*/GLU; 3) microalgal strains; 4) *A. fumigatus*/TWS-algal pellets; 5) *A. fumigatus*/GLU-algal pellets.

Fatty acids composition (measured by composition of FAMEs) of *A. fumigatus*/GLU and *A. fumigatus*/TWS pellets with microalgal species is shown in Figure 4. Fatty acids in fungi are represented mainly by palmitate (C16:0), stearate (C18:0), oleate, C18:1 and linoleate C18:2 [51], [47], [48], [52]. Fatty acid composition of *A. fumigatus*/GLU was also dominated by palmitate, C16:0 (ca 20%), oleate, C18:1 (ca 30%) and linoleate, C18:2 (ca 30%) (Figure 4). *A. fumigatus*/TWS pellets showed almost similar concentrations of palmitate (C16:0), stearate (C18:0) and oleate (C18:1) as *A. fumigatus*/GLU, but a higher concentration of palmitoleate (16:1) and linolenate (18:3) and lower concentration of linoleate (C18:2).

Microalgal representatives showed different profiles of fatty acids compositions. Fatty acids composition of the fungal-algal pellets obviously reflected the compositions in both fungal and algal strains and the efficiencies of their co-pelletization. In all cases, both *A. fumigatus* and microalgal strains contributed to the level of palmitate (C16). *A. fumigatus* was a main contributor of the oleate (18:1) and linoleate (C18:2). Some microalgae were the main contributors of the linolenate (C18:3).

### Swine wastewater as an alternative source of nutrients for fungal-microalgal pellets

**Figure 5:**
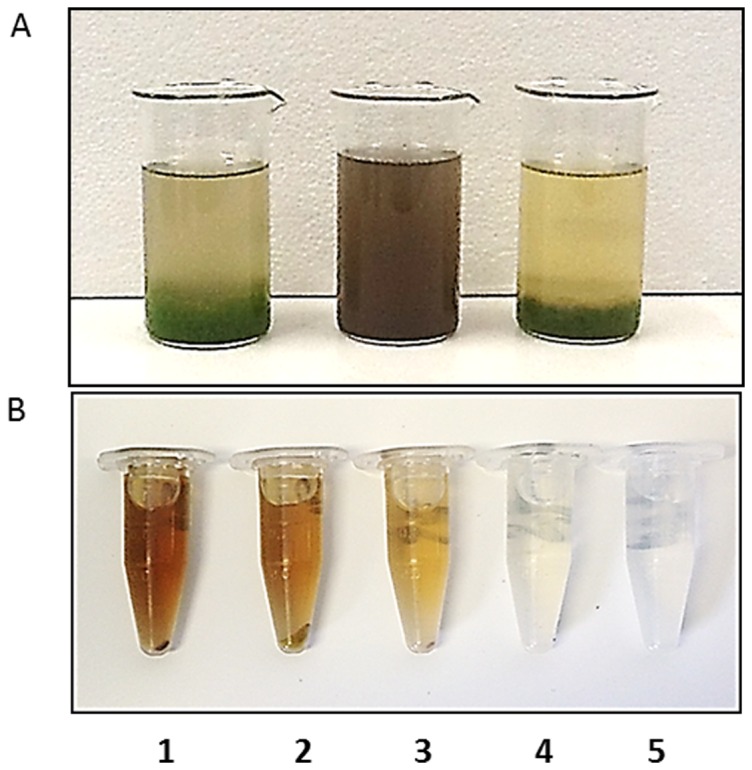
Application of *A. fumigatus*/*Thraustochytrid* sp and *A. fumigatus/T. chuii* pellets for 25% swine wastewater treatment. A) *A. fumigatus/Thraustochytrid* sp pellets (left) and *A. fumigatus/T. chuii* pellets (right) 48 hr after mixing with 25% swine wastewater. 25% swine wastewater at t = 0 (middle); B) samples of 25% wastewater before (1) and after treatment with *Thraustochytrid* sp (2), *A. fumigatus* (3) and *A. fumigatus/Thraustochytrid* sp (4). Tape water (5).

**Figure 6:**
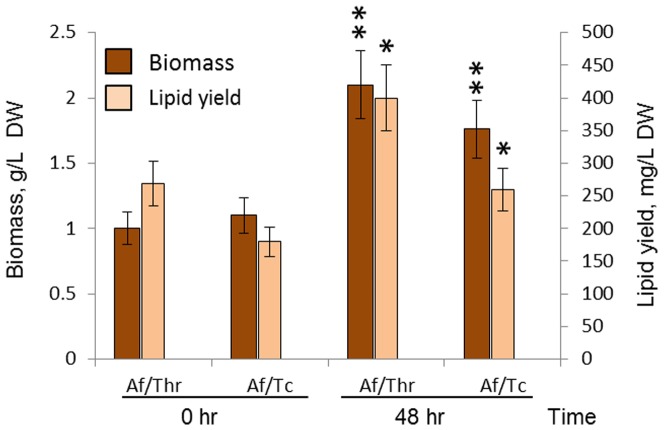
Biomass and lipid production in *A. fumigatus/Thraustochytrid* sp and *A. fumigatus/T. chuii* pellets grown in 25% swine wastewater. Af/Thr: *A. fumigatus*/*Thraustochytrid* sp pellets; Af/Tc: *A. fumigatus*/*T. chuii* pellets. *A. fumigatus*/microalgal co-cultures which showed synergistic effects with significance levels: *p<0.05; **p<0.01.

We assessed the ability of *A. fumigatus/Thraustochytrid* (Af/Thr) and *A. fumigatus/T. chuii* pellets (Af/Tc) pellets to uptake the main nutrients (NH_4_^+^-N and PO_4_^−3^-P) from diluted ASW prepared from swine lagoon wastewaters (Table 3, Figure 5). For these experiments, the swine wastewater was diluted to 10% and 25% with sterile seawater. After 48 h of Af/Thr incubation in 25% wastewater the concentration of NH_4_^+^-N was reduced from 164.3 mg/L to 22.2 mg/l (86% uptake) and the concentration of PO_4_^−3^-P was reduced from 38.7 mg/L to 11.8 mg/l (69% uptake). This removal efficiency was close to the additive efficiencies of NH_4_^+^-N, and PO_4_^−3^-P removal achieved separately by *Thraustochytrid* sp (30% and 18%, respectively) and *A. fumigatus* (43% and 31%, respectively) (Table 3). In 10% ASW both nutrients were almost compoletely removed after 48 h of incubation (96% removal for NH_4_^+^-N and 84% removal of PO_4_^−3^-P). Similar NH_4_^+^-N, and PO_4_^−3^-P removal efficiencies were observed after treatment of 25% and 10% ASW by Af/Tc pellets. Nutrient uptake by Af/Thr and Af/Tc pellets led to 2.1- and 1.6-fold increases in their biomass production after 48 h of treatment and the lipid yield increased by 1.4-fold for both pellets (Figure 6).

**Table 3:** Concentrations of nutrients in 25% swine wastewater before and after treatment with *Thraustochytrid* sp and *T. chuii* and their pellets with *A. fumigatus*.

ASW			*A. fumigatus*		*Thraustochytrid* sp		*Thraustochytrid* sp+*A. fumigatus*	
Concentration	NH_4_^−^, mg/l	PO_4_^−3^, mg/l	NH_4_^−^, mg/l	PO_4_^−3^, mg/l	NH_4_^−^, mg/l	PO_4_^−^,3 mg/l	NH_4_^−^, mg/l	PO_4_^−3^, mg/l
**ASW, 100%**	680.7±23.1	145.4±13.7	NA	NA	NA	NA	NA	NA
**ASW, 25%**	164.3±13.2	38.7±3.4	92.8±11.6	19.6±4.3	94.8±10.1	21.0±4.6	22.2±5.8	11.8±2.1
**ASW, 10%**	66.1±4.3	16.1±3.0	19.7±3.2	7.1±2.1	21.9±5.5	7.0±1.1	2.1±0.8	2.5±0.8

ASW			*A. fumigatus*		*T. chuii*		*T. chuii+A. fumigatus*	
Concentration	NH_4_^−^, mg/l	PO_4_^−3^, mg/l	NH_4_^−^, mg/l	PO_4_^−3^, mg/l	NH_4_^−^, mg/l	PO_4_^−3^, mg/l	NH_4_^−^, mg/l	PO_4_^−3^, mg/l
**ASW, 100%**	680.7±23.1	145.4±13.7	NA	NA	NA	NA	NA	NA
**ASW, 25%**	164.3±13.2	38.7±3.4	92.8±11.6	19.6±4.3	112.1±11.2	23.0±4.6	36.9±10.0	19.0±5.6
**ASW, 10%**	66.1±4.3	16.1±3.0	19.7±3.2	7.1±2.1	29.5±5.1	9.6±2.3	2.9±1.4	3.0±1.1

## Discussion

### *A. fumigatus*-mediated microalgal flocculation

Three microalgal features: small size (5∼30 µm), low concentration (0.02–0.05% DW) and negative surface charge make their large-scale harvesting the most costly step in biodiesel production. Fungal-assisted microalgal flocculation attracts attention because of its high harvesting efficiency. So far it has been shown for just one microalgal strain, the freshwater algae, *C. vulgaris*. Efficient flocculation of the *C. vulgaris* has been shown for a number of cultured filamentous fungal strains, including representatives of *Aspergilium* sp, *A. niger* and *A. oryze* [42], [22], [37], [53], [38], [41]. Our work found that *A. fumigatus* can efficiently flocculate a wide range of microalgal strains including marine and motile representatives. Lowest flocculation efficiency was found for the largest in size (300 µm) dinoflagellate, *P. lunula*. The detailed mechanisms of the fungal-algal interactions are still not clear. It was suggested that the algae have negative surface charge (−23.7 mV) due to of the presence of proton-active carboxylic, phosphoric, phosphodiester, hydroxyl and amine functional groups [19], [15]. Fungal hyphae and mycelia contain polysaccharides that were shown be positively charged (+46.1 mV) and therefore can potentially neutralize the negative charges on the algal surface, thereby enabling attachment to the fungal cell wall [54]. Microscopic examination of the algal cells in our study showed they bind to the fungal cells rather than get entrapped within the fungal filaments, potentially as a result of static interaction between opposite charged surfaces. The fact that no significant differences in harvesting efficiency were observed between non motile and motile *C. reinhardtii* and *T. chuii* suggests that this interaction may be strong.

### Effect of *A. fumigatus*-microalgal association on biomass and lipid production

Another attractive advantage of fungal-assisted flocculation is that both partners can contribute to the total biomass and lipid levels and compositions. As a result, the total biomasses of most fungal-algal pellets were found to be higher than the additive biomass of mono-cultured algal and fungal strains. Potential utilization of the cell wall carbohydrates by *A. fumigatus* can explain the synergistic effect of algal-fungal pelletization on total biomass enhancement. A correlation between enhanced biomass of the fungal-algal pellets and secretion of cell-wall degrading cellulases was also observed after co-cultivation of the filamentous fungi, *C. echinulatathis* with *C. vulgaris* [55]. In our experiments the potential cellulase activity correlated with the observation of cell wall-free microalgal protoplasts found either attached to the fungal cells or remained uncaptured in cultivation media. The saprophytic behaviour of the fungal component of natural lichens involving secretion of phenol oxidases, peroxidases, and cellulases benefit their growth when algal photosynthesis is limited [56].

Concentrations and total yield of lipids were not always correlated with the amount of generated total biomass after co-cultivation. This was most clearly seen after flocculation of microalgae with *A. fumigatus*/TSW pellets. Analysis of fatty acids showed both partners contribute to the fatty acids composition of the pelled biomasses. This suggests that fatty acids composition can be can be tailored and optimised using different sets of fungal and microalgal partners. Synergistic effects of co-cultivating *A. niger*, *A. oryzae* and *C. echinulata*, with *C. vulgaris* on biomass production and lipid yield has also been found by others [41], [42], [38], [22].

Mono-cultured algal and fungal cells have been extensively used for efficient recovery of the main nutrients, N and P and microelements including heavy metals from different types of wastewaters [57], [58], [27], [59], [9], [11], [60], [61]. *A. fumigatus/Thraustochytrid* and *A. fumigatus/T. chuii* pellets showed the additive effect on absorption rates of ammonium and phosphates from ASW diluted by seawater. Dilution of wastewater with seawater reduces the consumption of freshwater making the procedure more economical. Efficient wastewater treatment by fungal/algal pellets *Aspergillus sp/C. vulgaris* pellets has been previously shown by others [53], [42]. Wastewater with much lower concentrations of NH_4_^+^, though similar concentrations of PO_4_^3+^ (51.2 mg/l for both) was used in this study.

### Alternative carbon sources in fungal-assisted flocculation

In our study *A. fumigatus* grown on TWS as the sole source of carbon led to the production of *A. fumigatus*/TWS pellets which showed relatively high rates of trapping for most of the microalgal strains within the first 24 h. Lignocellulosic waste containing cellulose, hemicelluloses and lignin is one of the largest carbon sources that can be used as feedstock for large scale fungal biomass production. To convert cell wall polymers into reduced sugars fungal cells produce a cocktail of secreted hydrolytic enzymes, including cellulases, hemicellulases, pectinases, laccase manganese peroxidase and lignin peroxidase [62], [53], [41]. Cellulases are represented by three key enzymes, endoglucanases (EGs), cellobiohydrolases (CBHs) and β-glucosidases, which work synergistically to degrade the cellulose fraction [63]. Cellulolytic *A. fumigatus* Z5 growing in the presence of rice straw showed a induced endoglucanase, exoglucanase, β-glucosidase, laminarinase, lichenase, xylanase and pectin lyase activities [64]. The application of wheat straw biomass for fungal-assisted flocculation, however, needs to be optimised to reduce the production of anti-algal chemicals and to increase the lipid content in fungal cells grown on a straw biomass.

### Biofuel production from fungal-algal pellets

Fungal-algal biomass can be used for bio-diesel production through extraction of lipids followed by their transesterification (TE). In general, lipid extraction processes are energy intensive and costly since they involve expensive solvents and significant consumption of electricity [65], [66]. The following benchmark was proposed by US DOE for sustainable biofuel production from algae: the extraction process should consume (per day) no more than 10% of total energy produced (per day) (based on an algae energy content of 5 Wh/g) [67]. Some microalgal strains, such as *Nannochloropsis occulata* have very tough cell walls that require special pre-treatment for extraction of intracellular lipid [68]. Once extracted, lipids can be quantitatively transformed into biodiesel via TE technology with yields in excess of 98% (although, the use of algal oil in this process is a relatively new area of activity) [69].

Unlike microalgae the application of oleaginous fungi for biodiesel production is so far very limited in spite of obvious advantages over conventional plant and algal resources. Oleaginous fungi can accumulate over 20% (w/w) of their dry cell mass in the form of neutral lipids, with a high content of saturated and monounsaturated fatty acids, such as palmitic (C16:0), stearic (C18:0) and oleic (C18:1) commonly used for biodiesel production. Additional fungal features which makes them attractive feedstock for biofuel production includes their simple and fast growth rates in bioreactors unaffected by light intensity and their ability to utilize a wide range of lignocellulosic waste biomass as renewable carbon sources and wastewater nutrients as sources of nitrogen (N) and phosphorus (P) [70], [71], [57], [47]. Moreover, pelletization of fungal cells during growth makes their harvest much easier and cheaper than the isolation of microalgal strains (for review see [15]). The major limitation of fungal cells as feedstock for biofuel production is their tough cell walls which contains a complex structure composed of extensively cross-linked chitin, glucans and other polymers [72], [73].This may sharply increase the energy penalty for a large scale extraction procedure.

Pyrolysis, the thermal decomposition (400 to 550°C) of organic compounds in the absence of air/oxygen has recently attracted the increased attention of researchers due to a number of advantages, including relatively mild operational conditions and production of several valuable products: pyrolysis gas, bio-oil and bio-solids (bio-char and mineral ash). In most cases, bio-oil is a target product of pyrolysis because it could be further processed via catalytic hydrodeoxygenation (CHDO) and/or hydrocracking to liquid hydrocarbon products similar to petroleum-derived fuels. Recently, we reported pioneering studies on the pyrolysis of algal and aquatic plants representatives, which showed a great potential as a feedstock for the production of bio-oil and bio-char [74], [75], [66], [76], [77]. Some of these species were used for the efficient bioremediation of animal and mining wastewaters representing an attractive, ecologically friendly and potentially cost-effective solution for the conversion of waste biomass into sustainable bioenergy [75], [66]. In spite of the impressive biomass production rate, the high content of carbohydrates, proteins, lipids, and fatty acid composition there is no report on bio-oil production from the pyrolysis of oleaginous fungi. Considering the relatively low lipid content of fungi pyrolysis-CHDO seems to be more preferable route compared to extraction-TE. Detailed techno-economic evaluation of both approaches would be necessary to make a final determination of their economic feasibility and commercial viability.

## Conclusions

The described algal-fungal association shows a potential to solve a number of key challenges that algal biotechnology is facing, in particular:

- **Efficient harvesting of freshwater and seawater microalgae.** *A. fumigatus* showed efficient harvesting of 10 from 11 microalgal strains. Most of these strains are widely used by research groups and commercial companies for bio-diesel and value chemicals production. For the first time fungal-assisted flocculation was shown for marine microalgae.
- **Enhancement of total biomass, lipid production and optimization of fatty acids composition.** The additive and synergetic effects of *A. fumigatus* -algal pelletization on total biomass and lipid production were found for most of the microalgal strains. Our results showed that composition of fatty acids can be tailored and optimised through co-cultivating different algae and fungi without the need for genetic modification. Application of oleaginous fungal and algal strains can significantly improve total lipid yield.
- **Use of carbon, nitrogen and phosphorus from waste biomass as alternative, sustainable and renewable nutrient supply.** Use of alternative carbon and N and P sources along with subsequent wastewater purification can potentially improve the economics of large scale algal biotechnology. Dilution of wastewater with seawater reduced the amount of freshwater required, thereby making the whole process more economic.

## Supporting Information

Figure S1 **Lichen phenotypes.** Bar = 10 cm.
(TIF)

Figure S2 **Sedimentation of** ***A. fumigatus*** **/** ***T. chuii*** **pellets.**
(TIF)

Figure S3 **Microscopic analysis of** ***A. fumigatus*** **-miroalgal pellets.** A,B,M) *Thraustochytrid* sp; C,D) *D. tertriolecta*; E,F) *P. subcapitata*; G,H) *T. chuii*; I,J) *N. oculata*; K) *C. reinhardtii*; L) *P. lunula*; N) *A. fumigatus* filaments; O) *A. fumigatus/Thraustochytrid* sp pellets; P) *A. fumigatus/T. chuii* pellets. A,C,E,G,I,K,L: bright-field images; B,D,F,H,J: UV light images. Red spots represent chloroplast's fluorescence; M,N,O,P: Nile Red staining. Yellow spots represent oil bodies.; CV: cell walls. Scale = 50 µm.
(TIF)

Figure S4 **Evaluation of microalgal growth rates in the media containing 5% and 20% of** ***A. fumigatus*** **/TWS media.** A) Algal growth media containing 5% of *A. fumigatus*/TWS media; B) Algal growth media containing 20% *A. fumigatus*/TWS media.
(TIF)

Table S1 **Microalgal sources, characteristics and growth media.**
(XLSX)

## References

[1] Lam MK, Lee KT (2012) Microalgae biofuels: A critical review of issues, problems and the way forward. Biotechnology Advances 30:673–690. 22166620 10.1016/j.biotechadv.2011.11.008

[2] Olguin EJ (2012) Dual purpose microalgae-bacteria-based systems that treat wastewater and produce biodiesel and chemical products within a Biorefinery. Biotechnology Advances 30:1031–1046. 22609182 10.1016/j.biotechadv.2012.05.001

[3] Pinzi S, Leiva-Candia D, Lopez-Garcia I, Redel-Macias MD, Dorado MP (2014) Latest trends in feedstocks for biodiesel production. Biofuels Bioproducts & Biorefining-Biofpr 8:126–143.

[4] Wu YH, Hu HY, Yu Y, Zhang TY, Zhu SF, et al (2014) Microalgal species for sustainable biomass/lipid production using wastewater as resource: A review. Renewable & Sustainable Energy Reviews 33:675–688.

[5] Aguirre AM, Bassi A, Saxena P (2013) Engineering challenges in biodiesel production from microalgae. Critical Reviews in Biotechnology 33:293–308. 22804334 10.3109/07388551.2012.695333

[6] Rajkumar R, Yaakob Z, Takriff MS (2014) Potential of the Micro and Macro Algae for Biofuel Production: A Brief Review. Bioresources 9:1606–1633.

[7] Stephens E, Ross IL, Mussgnug JH, Wagner LD, Borowitzka MA, et al (2010) Future prospects of microalgal biofuel production systems. Trends in Plant Science 15:554–564. 20655798 10.1016/j.tplants.2010.06.003

[8] Wei P, Cheng LH, Zhang L, Xu XH, Chen HL, et al (2014) A review of membrane technology for bioethanol production. Renewable & Sustainable Energy Reviews 30:388–400.

[9] de Boer K, Moheimani NR, Borowitzka MA, Bahri PA (2012) Extraction and conversion pathways for microalgae to biodiesel: a review focused on energy consumption. Journal of Applied Phycology 24:1681–1698.

[10] Hamawand I, Yusaf T, Hamawand S (2014) Growing algae using water from coal seam gas industry and harvesting using an innovative technique: A review and a potential. Fuel 117:422–430.

[11] Duong VT, Li Y, Nowak E, Schenk PM (2012) Microalgae Isolation and Selection for Prospective Biodiesel Production. Energies 5:1835–1849.

[12] Borowitzka MA, Moheimani NR (2013) Sustainable biofuels from algae. Mitigation and Adaptation Strategies for Global Change 18:13–25.

[13] Leite GB, Abdelaziz AEM, Hallenbeck PC Algal biofuels: Challenges and opportunities. Bioresource Technology. 10.1016/j.biortech.2013.02.007 23499181

[14] Pragya N, Pandey KK, Sahoo PK (2013) A review on harvesting, oil extraction and biofuels production technologies from microalgae. Renewable and Sustainable Energy Reviews 24:159–171.

[15] Gultom SO, Hu B (2013) Review of Microalgae Harvesting via Co-Pelletization with Filamentous Fungus. Energies 6:5921–5939.

[16] Milledge J, Heaven S (2013) A review of the harvesting of micro-algae for biofuel production. Reviews in Environmental Science and Bio-Technology 12:165–178.

[17] Sharma KK, Garg S, Li Y, Malekizadeh A, Schenk PM (2013) Critical analysis of current microalgae dewatering techniques. Biofuels 4:397–407.

[18] Christenson L, Sims R (2011) Production and harvesting of microalgae for wastewater treatment, biofuels, and bioproducts. Biotechnology Advances 29:686–702. 21664266 10.1016/j.biotechadv.2011.05.015

[19] Molina Grima E, Belarbi EH, Acién Fernández FG, Robles Medina A, Chisti Y (2003) Recovery of microalgal biomass and metabolites: process options and economics. Biotechnology Advances 20:491–515. 14550018 10.1016/s0734-9750(02)00050-2

[20] Uduman N, Qi Y, Danquah MK, Hoadley AFA (2010) Marine microalgae flocculation and focused beam reflectance measurement. Chemical Engineering Journal 162:935–940.

[21] Leite GB, Abdelaziz AEM, Hallenbeck PC (2013) Algal biofuels: Challenges and opportunities. Bioresource Technology 145:134–141. 23499181 10.1016/j.biortech.2013.02.007

[22] Xia CJ, Zhang JG, Zhang WD, Hu B (2011) A new cultivation method for microbial oil production: cell pelletization and lipid accumulation by Mucor circinelloides. Biotechnology for Biofuels 4. 10.1186/1754-6834-4-15 PMC3127746 21635739

[23] Coward T, Lee JGM, Caldwell GS (2013) Development of a foam flotation system for harvesting microalgae biomass. Algal Research 2:135–144.

[24] Garg S, Wang LG, Schenk PM (2014) Effective harvesting of low surface-hydrophobicity microalgae by froth flotation. Bioresource Technology 159:437–441. 24690467 10.1016/j.biortech.2014.03.030

[25] Gonzalez-Fernandez C, Ballesteros M (2013) Microalgae autoflocculation: an alternative to high-energy consuming harvesting methods. Journal of Applied Phycology 25:991–999.

[26] Pires J, Alvim-Ferraz M, Martins F, Simoes M (2013) Wastewater treatment to enhance the economic viability of microalgae culture. Environmental Science and Pollution Research 20:5096–5105. 23673923 10.1007/s11356-013-1791-x

[27] Schenk PM, Thomas-Hall SR, Stephens E, Marx UC, Mussgnug JH, et al (2008) Second Generation Biofuels: High-Efficiency Microalgae for Biodiesel Production. Bioenergy Research 1:20–43.

[28] Vandamme D, Foubert I, Muylaert K (2013) Flocculation as a low-cost method for harvesting microalgae for bulk biomass production. Trends in Biotechnology 31:233–239. 23336995 10.1016/j.tibtech.2012.12.005

[29] Lee AK, Lewis DM, Ashman PJ (2013) Harvesting of marine microalgae by electroflocculation: The energetics, plant design, and economics. Applied Energy 108:45–53.

[30] Lee J, Cho DH, Ramanan R, Kim BH, Oh HM, et al (2013) Microalgae-associated bacteria play a key role in the flocculation of Chlorella vulgaris. Bioresource Technology 131:195–201. 23347927 10.1016/j.biortech.2012.11.130

[31] Manheim D, Nelson Y (2013) Settling and Bioflocculation of Two Species of Algae Used in Wastewater Treatment and Algae Biomass Production. Environmental Progress & Sustainable Energy 32:946–954.

[32] Powell RJ, Hill RT (2013) Rapid Aggregation of Biofuel-Producing Algae by the Bacterium Bacillus sp Strain RP1137. Applied and Environmental Microbiology 79:6093–6101. 23892750 10.1128/AEM.01496-13 PMC3811378

[33] Liu Z, Mutlib AE, Wang J, Talaat RE (2008) Liquid chromatography/mass spectrometry determination of endogenous plasma acetyl and palmitoyl carnitines as potential biomarkers of beta-oxidation in mice. Rapid Commun Mass Spectrom 22:3434–3442. 18837479 10.1002/rcm.3755

[34] Reed TB (1981) Biomass Gasification: Principles and Technology. Park Ridge, NJ Noyes Data Corporation.

[35] Guo S-L, Zhao X-Q, Wan C, Huang Z-Y, Yang Y-L, et al (2013) Characterization of flocculating agent from the self-flocculating microalga Scenedesmus obliquus AS-6-1 for efficient biomass harvest. Bioresource Technology 145:285–289. 23419992 10.1016/j.biortech.2013.01.120

[36] Wan C, Zhao X-Q, Guo S-L, Asraful Alam M, Bai F-W (2013) Bioflocculant production from Solibacillus silvestris W01 and its application in cost-effective harvest of marine microalga Nannochloropsis oceanica by flocculation. Bioresource Technology 135:207–212. 23218529 10.1016/j.biortech.2012.10.004

[37] Zhang JG, Hu B (2012) A novel method to harvest microalgae via co-culture of filamentous fungi to form cell pellets. Bioresource Technology 114:529–535. 22494571 10.1016/j.biortech.2012.03.054

[38] Luo SS, Dong ZJ, Wu XD, Liu YH, Ruan R (2013) Pelletization Behavior of Fungal Chlorella Sp Symbiosis System. Research Journal of Biotechnology 8:56–59.

[39] Xia CJ, Wei W, Hu B (2014) Statistical Analysis and Modeling of Pelletized Cultivation of Mucor circinelloides for Microbial Lipid Accumulation. Applied Biochemistry and Biotechnology 172:3502–3512. 24549800 10.1007/s12010-014-0759-8

[40] Liu Y, Liao W, Chen SL (2008) Study of pellet formation of filamentous fungi Rhizopus oryzae using a multiple logistic regression model. Biotechnology and Bioengineering 99:117–128. 17570715 10.1002/bit.21531

[41] Xie SX, Sun S, Dai SY, Yuan JS (2013) Efficient coagulation of microalgae in cultures with filamentous fungi. Algal Research-Biomass Biofuels and Bioproducts 2:28–33.

[42] Zhou WG, Min M, Hu B, Ma XC, Liu YH, et al (2013) Filamentous fungi assisted bio-flocculation: A novel alternative technique for harvesting heterotrophic and autotrophic microalgal cells. Separation and Purification Technology 107:158–165.

[43] Taylor TN, Hass H, Remy W, Kerp H (1995) The Oldest Fossil Lichen. Nature 378:244–244.

[44] Zoller S, Lutzoni F (2003) Slow algae, fast fungi: exceptionally high nucleotide substitution rate differences between lichenized fungi Omphalina and their symbiotic green algae Coccomyxa. Molecular Phylogenetics and Evolution 29:629–640. 14615198 10.1016/s1055-7903(03)00215-x

[45] Beckett RP, Zavarzina AG, Liers C (2013) Oxidoreductases and cellulases in lichens: Possible roles in lichen biology and soil organic matter turnover. Fungal Biology 117:431–438. 23809653 10.1016/j.funbio.2013.04.007

[46] Bushnell LD, Haas HF (1941) The utilization of certain hydrocarbons by microorganisms. Journal of Bacteriology 41:653–673. 16560430 10.1128/jb.41.5.653-673.1941 PMC374727

[47] Khot M, Kamat S, Zinjarde S, Pant A, Chopade B, et al (2012) Single cell oil of oleaginous fungi from the tropical mangrove wetlands as a potential feedstock for biodiesel. Microbial Cell Factories 11:71. 22646719 10.1186/1475-2859-11-71 PMC3442963

[48] Li SL, Feng SL, Li ZT, Xu H, Yu YP, et al (2011) Isolation, identification and characterization of oleaginous fungi from the soil of Qinghai Plateau that utilize D-xylose. African Journal of Microbiology Research 5:2075–2081.

[49] Lichtenthaler HK (1987) Chlorophylls and Carotenoids - Pigments of Photosynthetic Biomembranes. Methods in Enzymology 148:350–382.

[50] O'Fallon JV, Busboom JR, Nelson ML, Gaskins CT (2007) A direct method for fatty acid methyl ester synthesis: Application to wet meat tissues, oils, and feedstuffs. Journal of Animal Science 85:1511–1521. 17296772 10.2527/jas.2006-491

[51] Sergeeva YE, Galanina LA, Andrianova DA, Feofilova EP (2008) Lipids of filamentous fungi as a material for producing biodiesel fuel. Applied Biochemistry and Microbiology 44:523–527. 18822779

[52] Karimi K, Zamani A (2013) Mucor indicus: Biology and industrial application perspectives: A review. Biotechnology Advances 31:466–481. 23376652 10.1016/j.biotechadv.2013.01.009

[53] Zhou WG, Cheng YL, Li Y, Wan YQ, Liu YH, et al (2012) Novel Fungal Pelletization-Assisted Technology for Algae Harvesting and Wastewater Treatment. Applied Biochemistry and Biotechnology 167:214–228. 22538982 10.1007/s12010-012-9667-y

[54] Tan L, Qiu F, Lamport DTA, Kieliszewski MJ (2004) Structure of a hydroxyproline (Hyp)-arabinogalactan polysaccharide from repetitive Ala-Hyp expressed in transgenic Nicotiana tabacum. Journal of Biological Chemistry 279:13156–13165. 14724279 10.1074/jbc.M311864200

[55] Xie S, Sun S, Dai SY, S.Yuan J (2013) Efficient coagulation of microalgae in cultures with filamentous fungi. Algal Research 2:28–33.

[56] Webber MM, Webber PJ (1970) Ultrastructure of Lichen Haustoria - Symbiosis in Parmelia-Sulcata. Canadian Journal of Botany 48: 1521–&.

[57] Price MS, Classen JJ, Payne GA (2001) Aspergillus niger absorbs copper and zinc from swine wastewater. Bioresource Technology 77:41–49. 11211074 10.1016/s0960-8524(00)00135-8

[58] Swami D, Buddhi D (2006) Removal of contaminants from industrial wastewater through various non-conventional technologies: a review. International Journal of Environment and Pollution 27:324–346.

[59] Sharma P, Kaur H, Sharma M, Sahore V (2011) A review on applicability of naturally available adsorbents for the removal of hazardous dyes from aqueous waste. Environmental Monitoring and Assessment 183:151–195. 21387170 10.1007/s10661-011-1914-0

[60] Keymer P, Ruffell I, Pratt S, Lant P (2013) High pressure thermal hydrolysis as pre-treatment to increase the methane yield during anaerobic digestion of microalgae. Bioresource Technology 131:128–133. 23347920 10.1016/j.biortech.2012.12.125

[61] Singh AP, Singh T (2014) Biotechnological applications of wood-rotting fungi: A review. Biomass & Bioenergy 62:198–206.

[62] Kumar R, Singh S, Singh OV (2008) Bioconversion of lignocellulosic biomass: biochemical and molecular perspectives. Journal of Industrial Microbiology & Biotechnology 35:377–391. 18338189 10.1007/s10295-008-0327-8

[63] Krogh KBRM, Kastberg H, Jorgensen CI, Berlin A, Harris PV, et al (2009) Cloning of a GH5 endoglucanase from genus Penicillium and its binding to different lignins. Enzyme and Microbial Technology 44:359–367.

[64] Liu DY, Li J, Zhao S, Zhang RF, Wang MM, et al.. (2013) Secretome diversity and quantitative analysis of cellulolytic Aspergillus fumigatus Z5 in the presence of different carbon sources. Biotechnology for Biofuels 6. 10.1186/1754-6834-6-149 PMC3853031 24131596

[65] Muradov N (2014) Liberating Energy from Carbon: Introduction to Decarbonization: Springer New York Heidelberg Dordrecht London.

[66] Muradov N, Taha M, Miranda AF, Kadali K, Gujar A, et al.. (2014) Dual application of duckweed and azolla plants for wastewater treatment and renewable fuels and petrochemicals production. Biotechnology for Biofuels 7. 10.1186/1754-6834-7-30 PMC3944989 24576349

[67] DOE US (2010) National Algal Biofuels Technology Roadmap. US Department of Energy, Office of Energy Efficiency and renewable Energy, Biomass Program.

[68] Surendhiran D, Vijay M (2014) Effect of Various Pretreatment for Extracting Intracellular Lipid from Nannochloropsis oculata under Nitrogen Replete and Depleted Conditions. ISRN Chemical Engineering 2014:9.

[69] Sharif Hossain ABM, Salleh A, Boyce AN, Chowdhury P, Naqiuddin M (2008) Biodiesel Fuel Production from Algae as Renewable Energy. American Journal of Biochemistry and Biotechnology 4:250–254.

[70] Heredia-Arroyo T, Wei W, Hu B (2010) Oil Accumulation via Heterotrophic/Mixotrophic Chlorella protothecoides. Applied Biochemistry and Biotechnology 162:1978–1995. 20443076 10.1007/s12010-010-8974-4

[71] Bondioli P, Della Bella L, Rivolta G, Zittelli GC, Bassi N, et al (2012) Oil production by the marine microalgae Nannochloropsis sp F&M-M24 and Tetraselmis suecica F&M-M33. Bioresource Technology 114:567–572. 22459965 10.1016/j.biortech.2012.02.123

[72] Yin LJ, Jiang ST, Pon SH, Lin HH (2010) Hydrolysis of Chlorella by Cellulomonas sp YJ5 Cellulases and Its Biofunctional Properties. Journal of Food Science 75:H317–H323. 21535607 10.1111/j.1750-3841.2010.01867.x

[73] Wittenberg KM (1991) Preservation of High-Moisture Hay in Storage through the Use of Forage Additives. Canadian Journal of Animal Science 71:429–437.

[74] Ranzi E, Cuoci A, Faravelli T, Frassoldati A, Migliavacca G, et al (2008) Chemical Kinetics of Biomass Pyrolysis. Energy & Fuels 22:4292–4300.

[75] Miranda AF, Muradov N, Gujar A, Stevenson T, Nugegoda D, et al (2014) Application of Aquatic Plants for the Treatment of Selenium-Rich Mining Wastewater and Production of Renewable Fuels and Petrochemicals. Journal of Sustainable Bioenergy Systems 4:97–112.

[76] Muradov N, Fidalgo B, Gujar AC, Garceau N, T-Raissi A (2012) Production and characterization of Lemna minor bio-char and its catalytic application for biogas reforming. Biomass & Bioenergy 42:123–131.

[77] Muradov NZ, Veziroğlu TN (2008) “Green” path from fossil-based to hydrogen economy: An overview of carbon-neutral technologies. International Journal of Hydrogen Energy 33:6804–6839.

