# Supplementary figures and images for: Co-Cultivation of Fungal and Microalgal Cells as an Efficient System for Harvesting Microalgal Cells, Lipid Production and Wastewater Treatment
Source: PLoS One. 2014 Nov 24;9(11):e113497. doi: 10.1371/journal.pone.0113497 (PMC4242625; doi:10.1371/journal.pone.0113497)

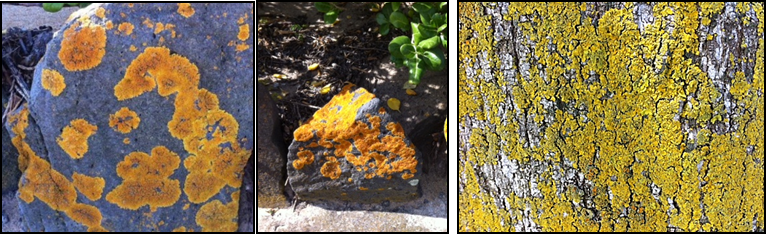

Supplement: Figure S1 — Lichen phenotypes. Bar = 10 cm. (TIF) [file pone.0113497.s001.tif]

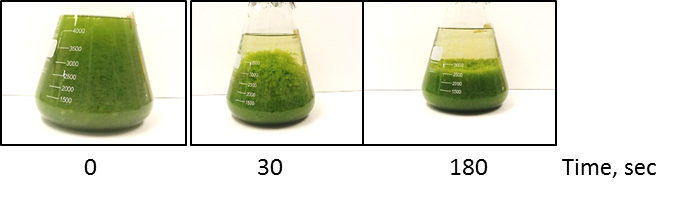

Supplement: Figure S2 — Sedimentation of A. fumigatus / T. chuii pellets. (TIF) [file pone.0113497.s002.tif]

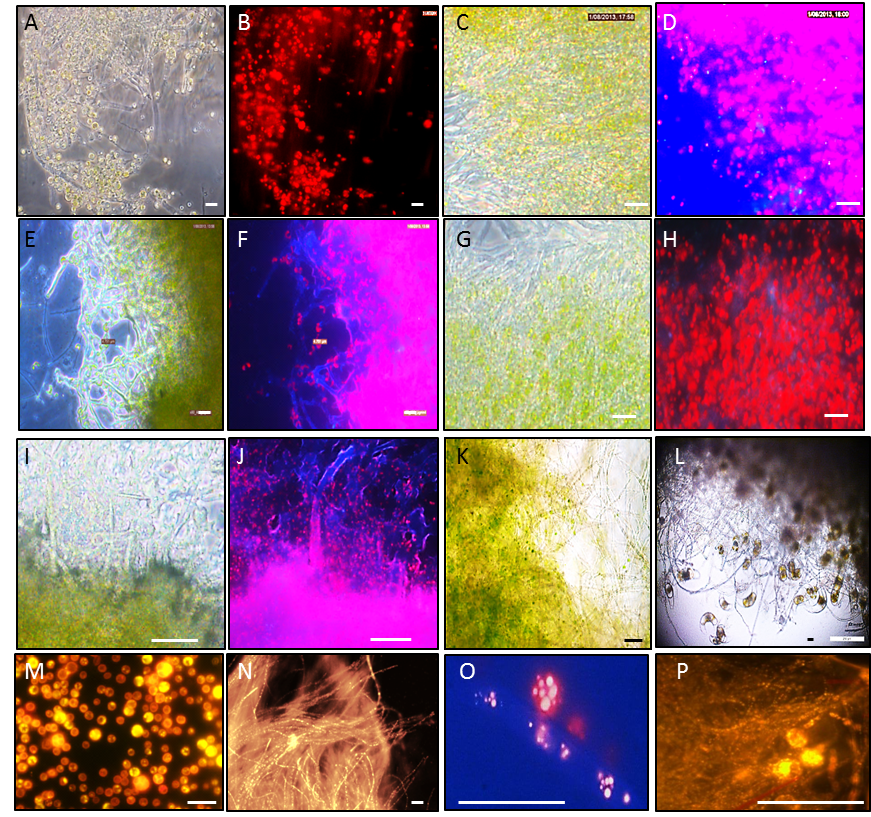

Supplement: Figure S3 — Microscopic analysis of A. fumigatus -miroalgal pellets. A,B,M) Thraustochytrid sp; C,D) D. tertriolecta; E,F) P. subcapitata; G,H) T. chuii; I,J) N. oculata; K) C. reinhardtii; L) P. lunula; N) A. fumigatus filaments; O) A. fumigatus/Thraustochytrid sp pellets; P) A. fumigatus/T. chuii pellets. A,C,E,G,I,K,L: bright-field images; B,D,F,H,J: UV light images. Red spots represent chloroplast's fluorescence; M,N,O,P: Nile Red staining. Yellow spots represent oil bodies.; CV: cell walls. Scale = 50 µm. (TIF) [file pone.0113497.s003.tif]

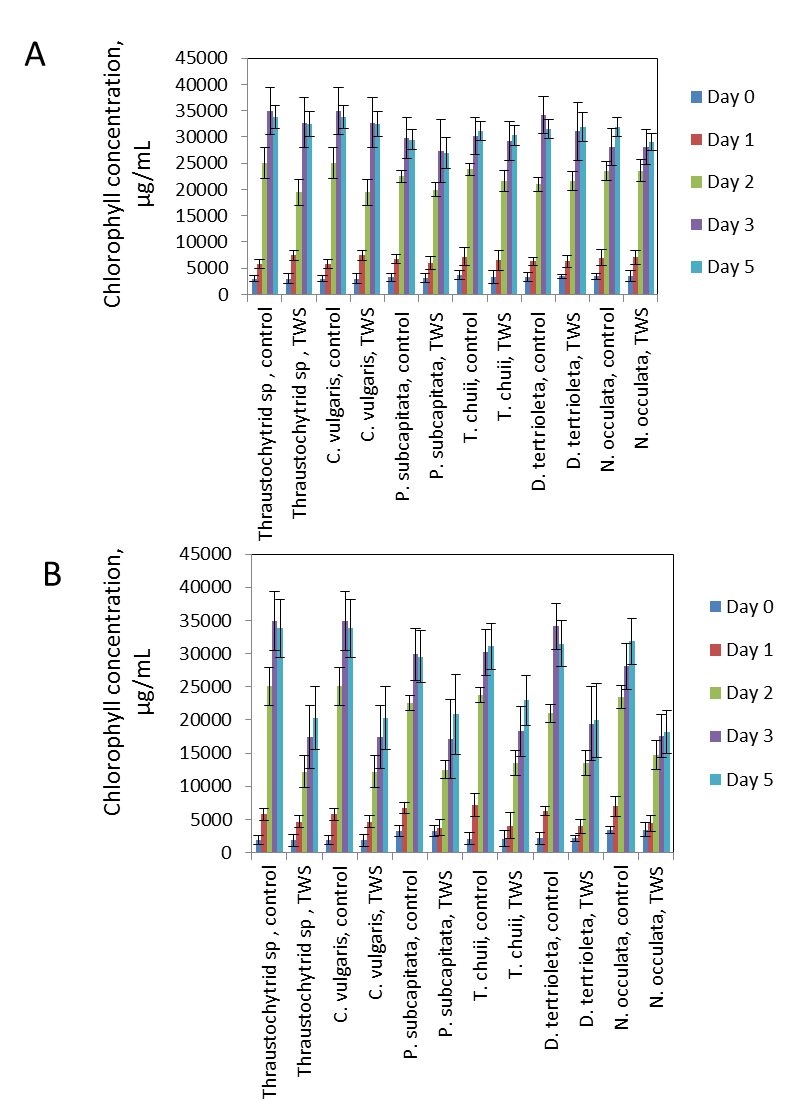

Supplement: Figure S4 — Evaluation of microalgal growth rates in the media containing 5% and 20% of A. fumigatus /TWS media. A) Algal growth media containing 5% of A. fumigatus/TWS media; B) Algal growth media containing 20% A. fumigatus/TWS media. (TIF) [file pone.0113497.s004.tif]
